# A Study on the Impact of Vanadium Doping on the Structural, Optical, and Optoelectrical Properties of ZnS Thin Films for Optoelectronic Applications

**DOI:** 10.3390/mi16030337

**Published:** 2025-03-14

**Authors:** H. Y. S. Al-Zahrani, I. M. El Radaf, A. Lahmar

**Affiliations:** 1 Physics Department, College of Science& Arts, King Abdulaziz University, Rabigh 21911, Saudi Arabia; 2 Department of Physics, College of Science, Qassim University, Buraydah 51452, Saudi Arabia; i.radaf@qu.edu.sa; 3 Electron Microscope and Thin Films Department, Physics Research Institute, National Research Centre, 33 El Bohoos Str., Dokki, Giza 12622, Egypt; 4 Laboratoire de Physique de la Matière Condensée (LPMC), Université de Picardie, Jules Verne, Pôle Scientifique, CEDEX 1, 80039 Amiens, France

**Keywords:** V-doped ZnS films, nebulizer spray pyrolysis, X-ray diffraction, energy gap, dispersion parameters, electrical conductivity

## Abstract

This study details the manufacture of vanadium-doped ZnS thin films via a cost-effective spray pyrolysis technique at varying concentrations of vanadium (4, 8, and 12 wt.%). The XRD data demonstrate the hexagonal structure of the vanadium-doped ZnS layers. The analysis of their structural properties indicates that the crystallite size (D) of the vanadium-doped ZnS films decreased as the vanadium concentration rose. The strain and dislocation density of the analyzed films were enhanced by increasing the vanadium content from 4 to 12 wt.%. The linear optical results of the vanadium-doped ZnS films revealed that the refractive index values were improved from 2.31 to 3.49 by increasing the vanadium concentration in the analyzed samples. Further, the rise in vanadium content enhanced the absorption coefficient. The energy gap (Eg) study indicates that the vanadium-doped ZnS films exhibited direct optical transitions, with the Eg values diminishing from 3.74 to 3.15 eV as the vanadium concentration increased. The optoelectrical analysis shows that the rise in vanadium concentration increases the dispersion energy from 9.48 to 12.76 eV and reduces the oscillator energy from 3.69 to 2.17 eV. The optical carrier concentration of these layers was improved from 1.49 × 1053 to 2.15 × 1053, while the plasma frequency was decreased from 4.34 × 1013 to 3.67 × 1013 by boosting the vanadium concentration from 4 to 12 wt.%. Simultaneously, the increase in vanadium content improves the nonlinear optical parameters of the vanadium-doped ZnS films. The hot probe method identifies these samples as n-type semiconductors. The findings suggest that these samples serve as an innovative window layer.

## 1. Introduction

Recently, chalcogenide materials based on sulfur have garnered significant interest due to their exceptional optical, optoelectronic, and electrical characteristics [1,2]. These chalcogenide semiconducting compounds exhibit elevated thermal stability and a high absorption coefficient [3,4,5]. The distinctive characteristics of these quaternary chalcogenides make them appropriate for several applications, including optical memory systems, absorber layers for solar cells, infrared sensors, and photodetectors. Previous articles have focused on chalcogenide films such as SnS, CdS, CuS, ZnS, In2S3, and MnS thin films due to their stability, affordability, ease of manufacturing, and abundance on Earth [6,7,8].

Zinc sulfide (ZnS), a critical semiconductor in the II-VI group, exhibits exceptional electrical and optical properties and a broad bandgap energy of 3.7 eV, attracting substantial interest [9,10]. ZnS has a high refractive index, making it a suitable candidate for antireflection coatings on commercial silicon solar cells. It has been meticulously analyzed for potential applications in various areas, including antireflection coatings, hydrogen production, blue-light diodes, window layers for solar cells, electroluminescent displays, optoelectronic devices, and various nonlinear optical devices [11,12]. Many articles have presented the fabrication of ZnS layers using chemical methods like sol–gel [13], spray pyrolysis [14], electrodeposition method [15], hydrothermal procedures [16], and simple chemical bath deposition [17]. J. Vidal et al. reported the deposition of ZnS layers on glass slides utilizing a chemical bath deposition procedure. The resulting layers were white, homogenous, and exhibited strong adhesion to the substrate. The results regarding the layers’ electrical and optical properties were influenced by the NH3 concentration and annealed temperature. They showed that the XRD of these layers is polycrystalline and these layers have a hexagonal structure. The increase in annealing temperature reduces the transmission and resistivity of these layers while enhancing their reflectance values [18].

Moreover, Dona et al. report the production of ZnS layers using a cost-effective chemical bath deposition procedure that utilizes NH_3_ and hydrazine (N2H_4_) as complexing agents. The XRD analysis revealed that these sheets have a cubic structure. An examination of their electron diffraction has demonstrated that the films display elevated stoichiometry. The ZnS layers produced by this method demonstrate a remarkable transmittance of above 75% [19]. On the other hand, Isaiah O. Oladeji and Lee Chow investigated the influence of ammonium salt on chemically produced zinc sulfide thin films. The thickness of the formed film increases when deposition occurs at room temperature and in the presence of an ammonium salt. The thickness of the produced thin film at ambient temperature was 93.5 nm; however, upon heating the water bath to 85 °C, the thickness was reduced to 20 nm. The transmission of the film in the visible spectrum, such as at 600 nm, is approximately 75%. The Eg of these zinc sulfide layers was determined to be 3.9 eV [20]. ZnS thin layers employed as buffer layers in thin film solar cells are typically produced via the simple chemical bath deposition method, with hydrazine hydrate utilized as a supplementary complexing agent. This material is exceedingly toxic; thus, substituting it with a less harmful alternative is advisable [21].

Prior studies have concentrated on improving ZnS’s electrical and optical characteristics by incorporating metal ions such as Eu, Co, Fe, Cd, Mo, and Cu into these films [22,23,24]. Mosavi et al. examined the impact of the Cadmium concentration on the structural and optical properties of electrodeposited ZnS layers. Boosting the Cd ratio in these layers elevated the refractive index of the ZnS samples and decreased their band gap energy values [25]. Shaili et al. showed that adding copper to ZnS layers improves their structural properties and reduces their optical band gaps as the dopant’s concentration increases [26]. Jebathew et al. examined ZnS layers’ photo-sensing, structural, and optical properties when doped with Sb, Sn, and Al [27]. Previous research has focused on ZnS thin films doped with metals including Cd, F, Cu, In, and Al; however, vanadium-doped ZnS films have yet to be studied. This study presents the fabrication of vanadium-doped ZnS films using spray pyrolysis. This study examines the impact of vanadium doping on the optoelectrical, structural, and optical properties of ZnS layers, aiming to create a window layer with beneficial physical attributes for photovoltaic applications.

## 2. Materials and Methods

### 2.1. Synthesis of Vanadium-Doped ZnS Films

**Figure 1:**
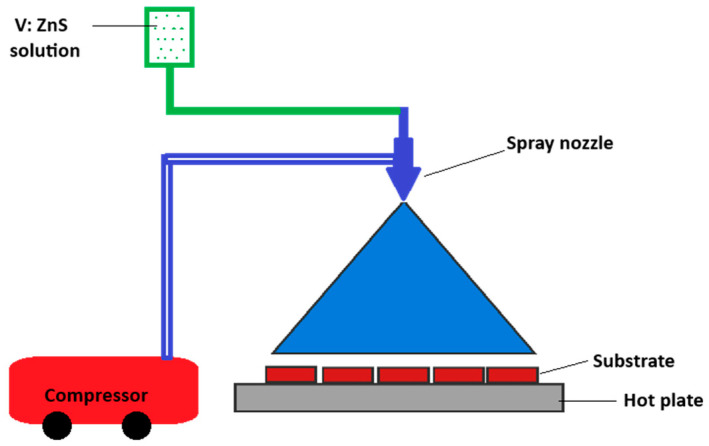
Schematic graph of the spray pyrolysis method.

Vanadium-doped ZnS films were deposited using a spray pyrolysis approach at 190 °C on glass substrates. We began preparing the vanadium-doped ZnS solution through the interaction of two solutions. The initial solution was formed by dissolving 0.2 M Zn (CH_3_COO)_2_ (zinc acetate, Sigma-Aldrich, Germany) in 50 mL of deionized water. The second solution was prepared by dissolving 0.2M CH_3_CSNH_2_ (thioacetamide, Sigma-Aldrich, Germany) in 50 mL of deionized water. All these chemicals had a purity of 99.99% and were brought from Sigma Aldrich. The solutions were combined sequentially, beginning with adding the zinc acetate solution to the thioacetamide solution. Their combination was thoroughly agitated at 60 °C for 60 min until a crimson solution was obtained. Subsequently, various ratios of ammonium vanadate (4, 8, and 12 wt.%) were added to the mixture to produce vanadium-doped ZnS films. The pH of the vanadium-doped ZnS solution was 8. The vanadium-doped ZnS solution was sprayed over a glass substrate pre-heated to 190 °C using the spray pyrolysis procedure, as depicted in Figure 1. The preparation conditions were meticulously regulated: the solution flow rate was 10 mL/min and the substrates employed were high-quality microscopic glass slides.

### 2.2. Characterization of Vanadium-Doped ZnS Layers

The thickness of the undoped and vanadium-doped ZnS layers was measured using a stylus mechanical profilometer, the Alpha Step model D-500 stylus. The crystal structure of the ZnS and vanadium-doped ZnS films was meticulously analyzed by a Philips X’Pert X-ray diffractometer from Holland, which utilized Cu-K*α* radiation with a wavelength of 1.54184 Å. The surface morphology of the vanadium-doped ZnS samples was analyzed by the Quanta model FeG-250 field-emission scanning electron microscope from the USA. The optical properties of the V: ZnS layers were determined by measuring the *T* and *R* using a Shimadzu (UV-3600) spectrophotometer from Jaban.

## 3. Results and Discussion

### 3.1. Structural Investigation

**Figure 2:**
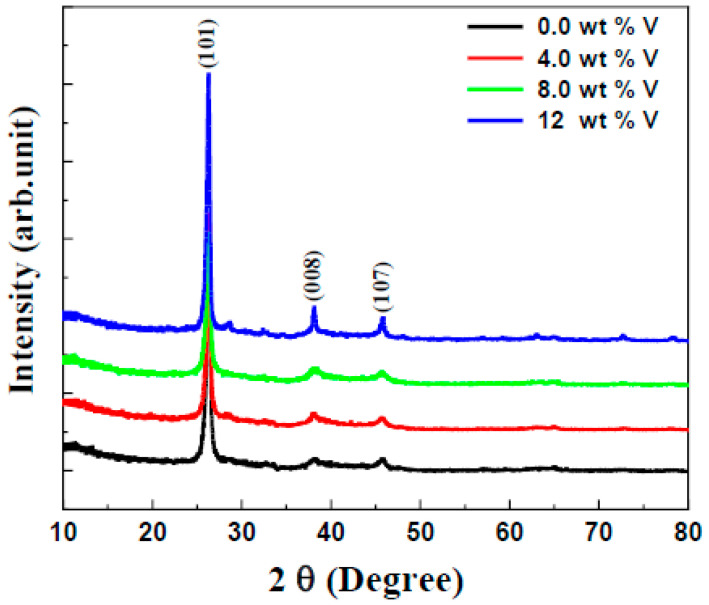
XRD patterns of the undoped and vanadium-doped ZnS layers.

In this study, X-ray diffraction (XRD) was exploited to determine the crystallographic structure of the vanadium-doped ZnS layers. Figure 2 displays the XRD patterns of the vanadium-doped ZnS layers formed with different vanadium contents that were obtained in the region of 2*θ* = 10–80°. Figure 1 illustrates that all film samples exhibit a single polycrystalline phase characterized by a hexagonal crystal structure. These results agree with those of previous articles [28]. The observed diffraction lines include (101), (008), and (107) at diffraction angles of 27.38°, 38.14°, and 44.95°, respectively. The line diffractions at these angles are consistent with the JCPDS No. 89-2739. The XRD card confirms that no additional phases were obtained, indicating that all samples exhibit a single-phase hexagonal structure. As the vanadium content increases, the 2θ is shifted to the right. This shift can be attributed to the substitution of Zn^2+^ ions with V^3+^ ions. The vanadium’s ionic radius (0.64 Å) is lower than the zinc’s ionic radius (0.74 Å). The smaller ionic radius of V^3+^ introduces a compressive strain and reduces the interplanar spacing (d-spacing) in the crystal structure. The increase in 2*θ* led to the observed rightward shift in the XRD peaks. This behavior is consistent with Vegard’s law and confirms the successful incorporation of vanadium into the ZnS lattice. Furthermore, the peak shift indicates the presence of lattice distortion and strain, which are typical characteristics of doped semiconductor systems.

This work uses Debye–Scherer relationships to determine the strain function (*ε*) and crystallite size (*D*) of the ZnS and vanadium-doped ZnS films [29,30]:

(1) $$D=\frac{0.9\;\lambda }{\beta COS\;\theta }$$

(2) $$\varepsilon =\frac{\beta COS\;\theta }{4}$$

In these formulas, *λ* refers to the X-ray wavelength (*λ* = 1.54 Å), *β* represents the full width at half maximum (FWHM), and *θ* denotes the Bragg diffraction angle.

Furthermore, we can estimate the number of crystallites ($N_{C}$) and the dislocation density (*δ*) of the vanadium-doped ZnS films by applying the following equations [31,32]:

(3) $$N_{C}=\frac{t}{D^{3}}$$

(4) $$\delta =\frac{1}{D^{2}}$$

**Figure 3:**
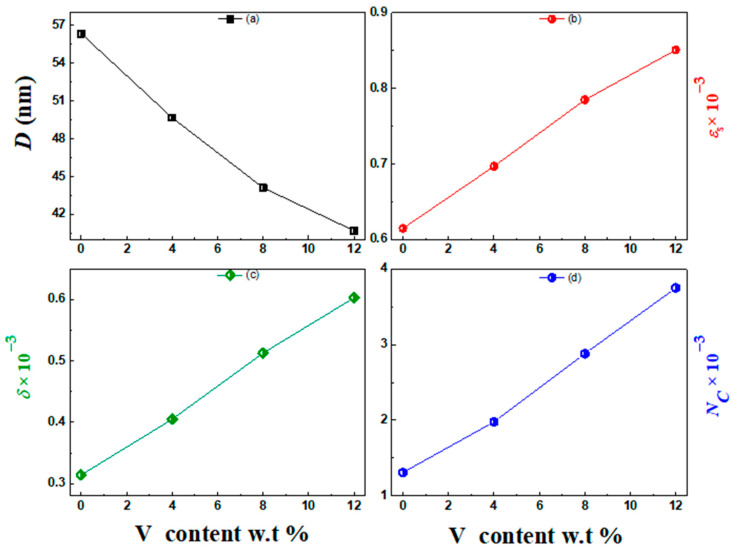
Structural indices of the undoped and vanadium-doped ZnS layers: (**a**) D vs. V content, (**b**) $\varepsilon_{s}\;vs.\;V$ content, (**c**) δ vs. V content, and (**d**) $N_{C}$ vs. V content.

The structural constants of the vanadium-doped ZnS layers are documented in Table 1. According to this table, the boost in vanadium content from 4 to 12 wt.% enhanced the $\varepsilon_{s}$, $N_{C}$, and *δ*, while the crystallite size values were minimized. The reduction in crystallite size can be attributed to the incorporation of V^3+^ ions into the ZnS lattice, which are substituted for Zn^2+^ ions due to the vanadium’s ionic radius being smaller than the zinc’s ionic radius. This substitution introduces lattice strain and defects, which disrupt the regular crystal structure and act as nucleation sites, promoting the formation of smaller crystallites. Additionally, the presence of V induces compressive stress and lattice distortion, further inhibiting the growth of larger crystallites. The variation in the structural indices of the ZnS and vanadium-doped ZnS layers with the vanadium content is presented in Figure 3. This plot refers to the reduction in the crystallite size values caused by boosting the vanadium content. In contrast, the $\varepsilon_{s}$, $N_{C}$, and *δ* values were enhanced.

### 3.2. Microstructural Study

**Figure 4:**
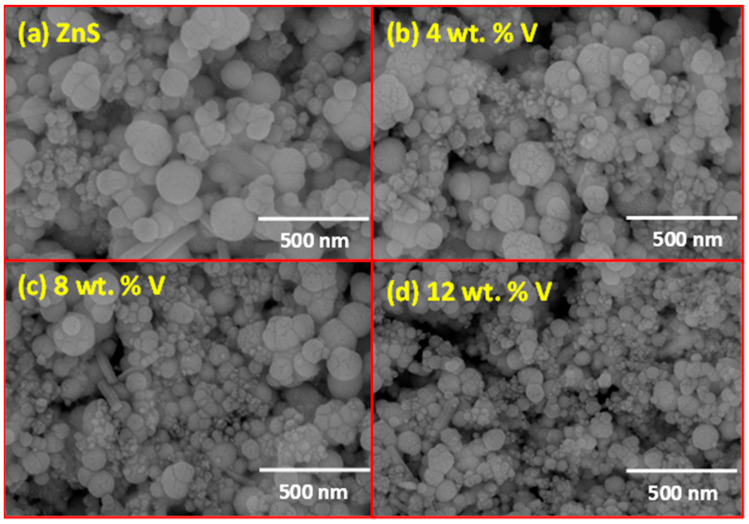
FESEM images of the ZnS and vanadium-doped ZnS layers: (**a**) ZnS, (**b**) 4 wt. % V, (**c**) 8 wt. % V, and (**d**) 12 wt.% V.

The surface morphology of the undoped and vanadium-doped ZnS layers was examined using a field emission scanning electron microscope (FESEM) from the USA. Figure 4a illustrates an FESEM image of the undoped ZnS film, revealing a smooth and uniform surface with well-defined grain boundaries, indicating a high degree of crystallinity and homogeneity. Furthermore, Figure 4b shows the ZnS film doped with 4 wt.% vanadium. The addition of 4 wt.% vanadium to the ZnS lattice decreases the grain size slightly, and the surface becomes more densely packed. This trend, related to the incorporation of V^3+^ ions into the ZnS lattice, introduces lattice strain and defects, leading to the formation of finer grains. The surface also begins to show a slight increase in roughness. On the other hand, Figure 4c displays the ZnS film doped with 8 wt.% vanadium. The grain size further decreases, and the surface becomes more textured. The grains are more densely packed, and the surface roughness is more pronounced. This is consistent with the increased lattice distortion and strain caused by higher vanadium concentrations, which inhibits the growth of larger crystallites. Meanwhile, Figure 4d depicts the ZnS film doped with 12 wt.% vanadium.

In this figure, the grains are very fine and densely packed, and the surface roughness is markedly increased. The presence of small aggregates or clusters is also observed, which may be the result of the segregation of vanadium at grain boundaries or the formation of secondary phases. This indicates that at higher doping levels, the vanadium significantly disrupts the regular crystal structure, leading to a more complex and rough surface morphology. These morphological changes are consistent with the XRD results, which show a reduction in crystallite size with increased vanadium doping. The enhanced surface roughness and finer grain structure in the V-doped films are expected to influence their optical and electrical properties, as these features can affect light scattering and charge carrier transport.

**Figure 5:**
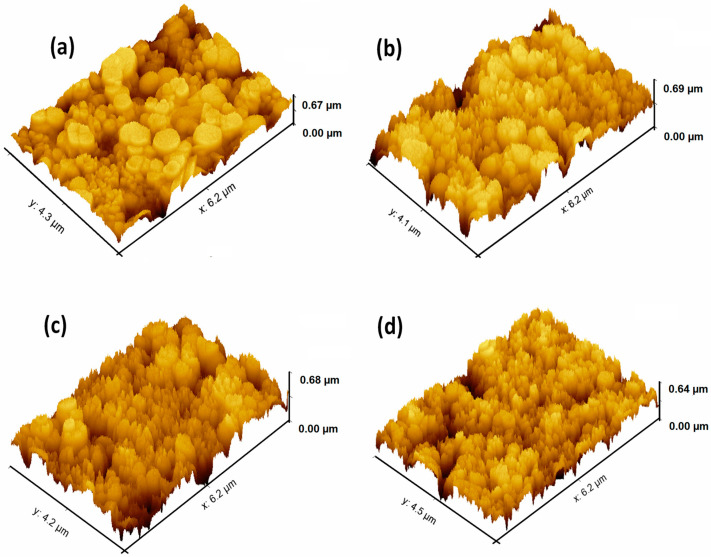
AFM images of the surfaces of the ZnS and vanadium-doped ZnS layers: (**a**) ZnS, (**b**) 4 wt. % V, (**c**) 8 wt. % V, and (**d**) 12 wt. % V.

The atomic force microscopy images of the undoped and vanadium-doped ZnS layers are presented in Figure 5a–d. The AFM image of the undoped ZnS film (Figure 5a) shows a relatively smooth and uniform surface with minimal roughness, consistent with a high degree of crystallinity and homogeneity. When increasing the vanadium doping to 4 wt% (Figure 5b), the surface begins to show slight variations in height and increased roughness. The grains appear more defined, and the surface starts to exhibit a textured morphology, indicating the initial impact of the vanadium incorporation on the film’s surface structure. At 8 wt. % V doping (Figure 5c), the surface roughness becomes more pronounced. The AFM image reveals a more complex topography, with higher peaks and deeper valleys. This increased roughness is consistent with the finer grain structure observed in the 2D SEM images, which is due to the lattice strain and defects introduced by higher vanadium concentrations. The film with 12 wt.% V doping (Figure 5d) exhibits the most significant surface roughness. The AFM image shows a highly textured surface with numerous peaks and valleys, indicating a complex and irregular morphology. This is likely due to the extensive lattice distortion and the presence of small aggregates or clusters caused by the high vanadium doping level.

**Figure 6:**
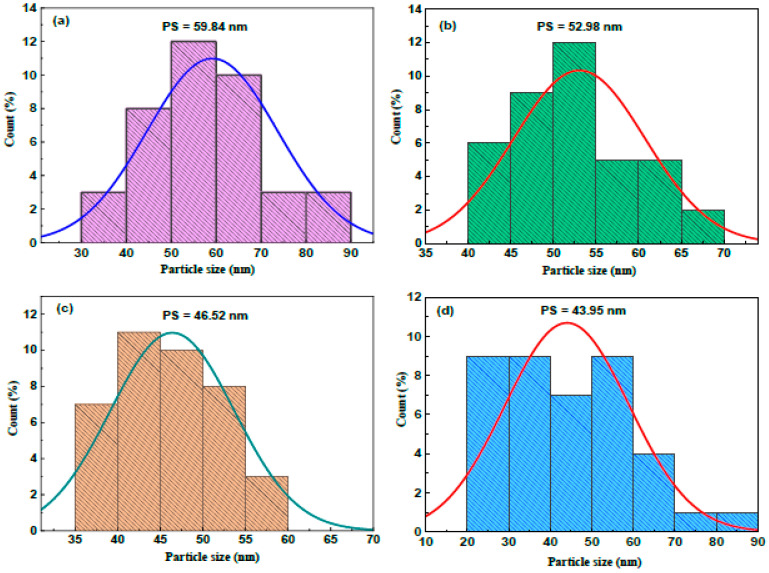
Particle size histograms of the ZnS and vanadium-doped ZnS layers: (**a**) ZnS, (**b**) 4 wt. % V, (**c**) 8 wt. % V, and (**d**) 12 wt. % V.

Figure 6a–d display the particle size (PS) histograms of the undoped and vanadium-doped ZnS films. The boost in vanadium content from 4 to 12 wt.% reduces the average PS from 59.84 nm to 43.95 nm.

### 3.3. Linear Optical Properties

**Figure 7:**
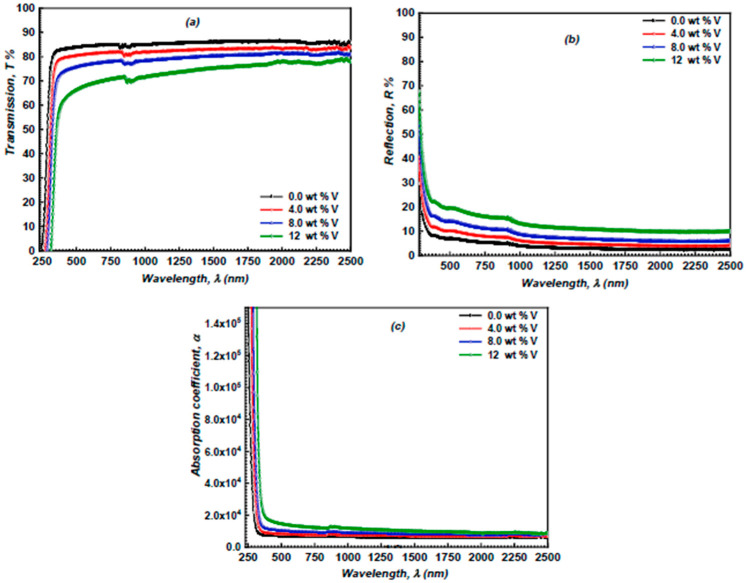
(**a**) The *T* of the ZnS and vanadium-doped ZnS films versus λ; (**b**) The *R* of the undoped and vanadium-doped ZnS layers versus *λ*; and (**c**) The α of the undoped and vanadium-doped ZnS films vs. *λ*.

The linear optical characteristics of the examined layers were estimated by recording the optical transmittance (*T*) and reflectance (*R*) spectra of the vanadium-doped ZnS films using a double-beam spectrophotometer. The T and R spectra of the vanadium-doped ZnS layers at normal incidence in the 250–2500 nm wavelength range are illustrated in Figure 7a,b. It has been observed that an increase in vanadium content results in a decrease in transmission. This reduction may be attributed to the incorporation of vanadium into the ZnS lattice, which introduced defects and lattice distortions, which worked as scattering centers for photons. As the doping concentration increases, the defect density rises, leading to greater light scattering and lower transmittance. Moreover, the increase in the vanadium content enhances the reflectance value of these films. This performance was related to the vanadium doping leading to an increase in surface roughness, as observed in the SEM and 3D SEM images. Rougher surfaces cause a more diffuse reflection of incident light, which increases the overall reflectance of the films.

The absorption coefficient denotes the degree to which light of a particular wavelength can infiltrate a material before absorption. A material with a low absorption coefficient will demonstrate little light absorption, resulting in a sufficiently thin specimen appearing transparent at a particular wavelength. The absorption coefficient depends on the material and the wavelength of the absorbed light. Semiconductor materials possess a notable advantage in their absorption coefficient, as photons with energy below the band gap do not have adequate energy to elevate an electron from the valence band to the conduction band. The absorption coefficient (α) of the ZnS and vanadium-doped ZnS layers can be determined by the following formula [33,34]:

(5) $$\alpha =\frac{1}{t}ln\left[\frac{{\left(1-R\right)}^{2}}{2T}+{\left(\frac{{\left(1-R\right)}^{4}}{{4T}^{2}}+R^{2}\right)}^{1/2}\right]$$

The fluctuation of the α of the ZnS and vanadium-doped ZnS samples in terms of λ is illustrated in Figure 7c. The increase in the vanadium concentration enhances the *α* of the examined samples. This performance can be related to the vanadium doping increasing the free carrier concentration in the ZnS layer. These free carriers can absorb photons, particularly in the infrared region, leading to an improvement in the absorption coefficient. The energy gap (*E_g_*) of the vanadium-doped ZnS samples was computed using Tauc’s formula [35,36]:

(6) $$\alpha h\nu =W{\left(h\nu -E_{g}\right)}^{x}$$

Here, *x* identifies the type of optical transition in the investigated films. Indirect optical transition occurred at *x* = 2, and direct optical transition occurred at *x* = 0.5.

**Figure 8:**
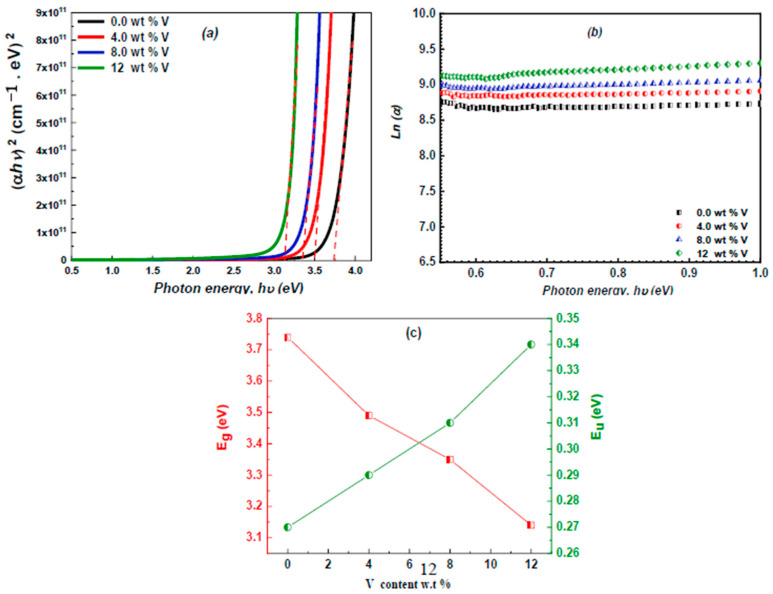
(**a**) The *E_g_* curve of the vanadium-doped ZnS films; (**b**) the Urbach curve of the undoped and vanadium-doped ZnS layers; and (**c**) the plot of the influence of the vanadium content on the *E_g_* and *E_u_*.

The *E_g_* of the ZnS and vanadium-doped ZnS samples can be estimated by plotting the relation between the (*αhν*)^2^ and *hv*, and the *E_g_* values equal the intercept of this plot, as presented in Figure 8a. The values of the *E_g_* of the vanadium-doped ZnS samples are documented in Table 2. The augmentation of the vanadium content diminished the *E_g_* of these layers from 3.74 to 3.15 eV due to the production of localized states concomitant with the elevated vanadium content [37,38]. The values of the energy gap in our work are near to the *E_g_* values reported by Rana et al. [39].

Meanwhile, the Urbach relationship was applied to estimate the Urbach energy (*E_u_*) of the vanadium-doped ZnS films [40,41]:

(7) $$\alpha =\alpha_{o}exp\left(h\upsilon /E_{u}\right)$$

The plot of ln (α) of the vanadium-doped ZnS layers versus *hv* is illustrated in Figure 8b. The graph’s slope was utilized to calculate the Eu values documented in Table 2. The augmentation of the vanadium content enhances the Eu of the analyzed layers due to the proliferation of localized states concomitant with the elevated vanadium ratio [42]. Figure 8c depicts the variance of *E_g_* and *E_u_* with changes in the vanadium content. It was observed that when the vanadium concentration grew, the bandgap energy (*E_g_*) fell and the Urbach energy (*E_u_*) boosted.

Furthermore, the extinction coefficient, *k*, is a critical parameter that unequivocally characterizes the interactions between the electric field component of the incident electromagnetic radiation and the film’s composition. During light propagation, electromagnetic wave scattering and amplitude or intensity losses result in diminished velocity. The k-values signify losses or decay, reflecting the attenuation of the oscillation amplitude of the electric field component of electromagnetic waves and forming the imaginary part of the refractive index. The extinction coefficient, *k*, of the vanadium-doped ZnS films, was evaluated using the following Equation [42,43]:

(8) $$k=\frac{\alpha \lambda }{4\pi }$$

**Figure 9:**
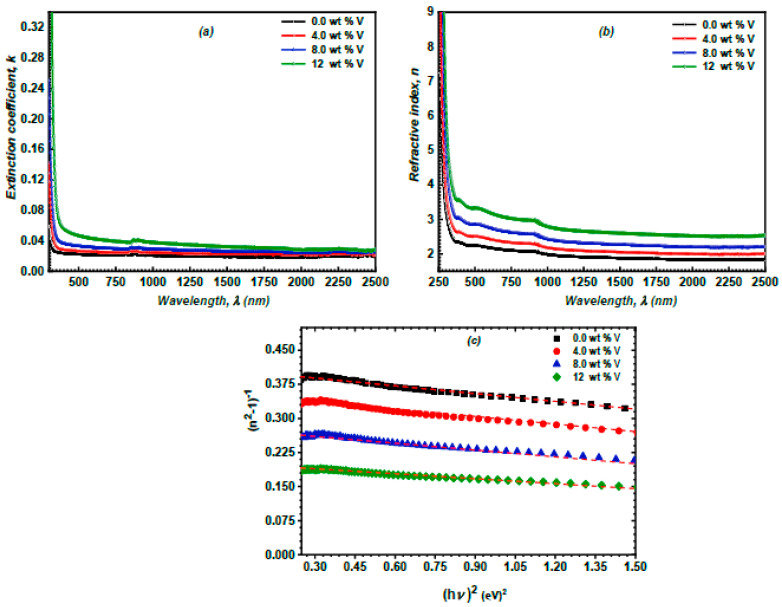
(**a**) The n of the spray-deposited vanadium-doped ZnS layers vs. *λ*; (**b**) the k of the spray-deposited vanadium-doped ZnS layers vs. *λ*; and (**c**) the plot of $(n^{2}-1{)}^{-1}$ against $(\hbar \upsilon {)}^{2}$ for the spray-deposited ZnS and vanadium-doped ZnS layers.

Figure 9a illustrates the *k* values of the vanadium-doped ZnS layers as a function of *λ*. The increase in the vanadium ratio enhances the k of the examined layers.

The refractive index (*n*) is an important optical parameter that represents the ratio of the velocity of light in a vacuum to its speed in a denser medium. As the refractive index of a material increases, the extent of the bending (or refraction) of a light beam upon entering or exiting the material similarly escalates. The refractive index (*n*) of the ZnS and vanadium-doped ZnS films was calculated by the following equation [44,45]:

(9) $$n=\frac{\left(1+R\right)}{\left(1-R\right)}-{\left(\frac{4R}{{\left(1-R\right)}^{2}}k^{2}\right)}^{\frac{1}{2}}$$

Figure 9b depicts the spectrum fluctuation of the n of the ZnS and vanadium-doped ZnS samples versus λ. Increasing the vanadium ratio enhanced the n of the examined layers. This performance is the result of enhancing the R values, which is achieved by increasing the vanadium content [46].

The oscillator energy (*E_o_*) and the dispersion energy (*E_d_*) are important dispersion parameters. The oscillator energy (*E_o_*) represents the average energy gap associated with electronic transitions in the material. The dispersion energy (*E_d_*) is a measure of the strength of interband optical transitions and is related to the material’s bonding and structural properties. It provides information about the density of states and the coordination number of atoms in the lattice. The dispersion indices of the spray-deposited ZnS and vanadium-doped ZnS samples, including their oscillator energy (*E_o_*) and dispersion energy (*E_d_*), have been derived from the Wemple–DiDomenico model based on the following expression [47,48]:

(10) $$n^{2}=1+\frac{E_{o}E_{d}}{E_{o}^{2}-{\left(h\upsilon \right)}^{2}}$$

Figure 9c depicts the correlation between $(n^{2}-1{)}^{-1}$ and $(h\upsilon {)}^{2}$ for the spray-deposited ZnS and vanadium-doped ZnS layers. This plot demonstrates that the values of *E_o_* and Ed can be obtained from the slope and intercept of the fitted linear equations. An increase in the vanadium content elevates the *E_d_* values. This increase can be attributed to vanadium doping, which introduces additional electronic states and defects into the ZnS lattice. These states increase the polarizability of the material, which is a key factor in determining the dispersion energy. Higher polarizability leads to stronger interactions between the electromagnetic field and the material, resulting in increased dispersion energy. Further, the rise in vanadium doping diminishes the *E_o_* values, as illustrated in Table 2. This trend is related to vanadium doping, which led to a narrowing of the bandgap of the investigated films. This narrowing results in lower energy transitions, which directly reduces the oscillator energy.

Moreover, the oscillator parameters of the spray-deposited ZnS and vanadium-doped ZnS layers were evaluated using the following equations [49,50]:

(11) $$f=E_{o}E_{d}$$

(12) $$n_{o}=\sqrt{1+\frac{E_{d}}{E_{o}}}$$

(13) $$\varepsilon_{s}={n_{o}}^{2}$$

In these formulas, *f* represents the oscillator strength, $\varepsilon_{s}$ denotes the static high-frequency dielectric constant, and no is the static refractive index value. 

Table 3 presents the analyzed magnitudes of the $\varepsilon_{s}$, *n_o_*, and *f* for the investigated layers. The boost in the vanadium ratio increases the values of $\varepsilon_{s}$ *n_o_*, and *n_o_* in the vanadium-doped ZnS films.

### 3.4. Nonlinear Optical Study

Investigating nonlinear optical properties is essential as it indicates the potential applications of semiconductor materials in switching devices, high-capacity communication systems, optical circuits, and photonic applications. The nonlinear optical parameters of the spray-deposited ZnS and vanadium-doped ZnS layers were assessed using Miller’s formulas [51,52]:

(14) $$\chi^{\left(1\right)}=\frac{{n_{0}}^{2}-1}{4\pi }$$

(15) $$\chi^{\left(3\right)}=B{\left[\frac{{n_{0}}^{2}-1}{4\pi }\right]}^{4}$$

(16) $$n_{2}=\frac{12\pi \chi^{\left(3\right)}}{n_{0}}$$

**Figure 10:**
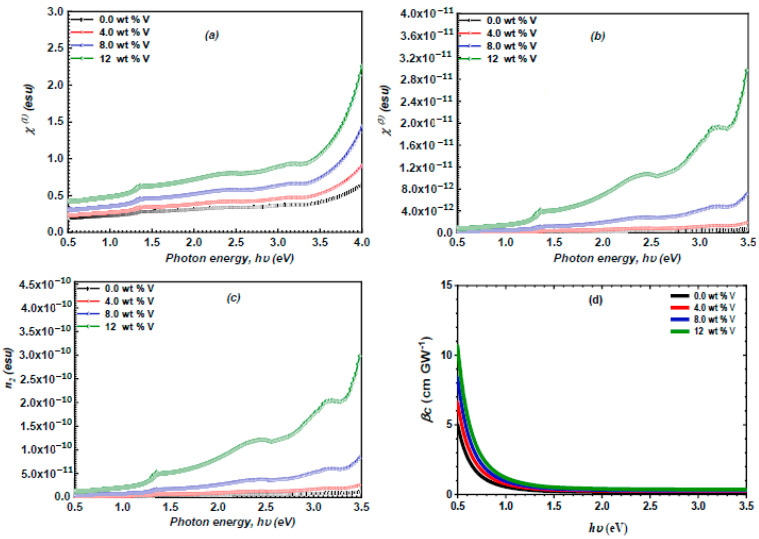
(**a**–**d**): the $\chi^{\left(1\right)}$, $\beta_{c},\;\chi^{\left(3\right)}$, and $n_{2}$ of the ZnS and vanadium-doped ZnS samples in terms of hν.

Figure 10a–d demonstrate the dependence of the nonlinear properties of the spray-deposited ZnS and vanadium-doped ZnS layers on the hv. The graphs demonstrate that the increase in vanadium content leads to an enhancement of the $\chi^{\left(1\right)}$, $\chi^{\left(3\right)}$, and $n_{2}$. The observed trend may be linked to vanadium doping producing additional electronic states and defects within the ZnS lattice. These states increase the polarizability of the material, which is a key factor in determining its nonlinear refractive index. Higher polarizability leads to stronger interactions with the electromagnetic field, resulting in an increased nonlinear refractive index. Furthermore, the magnitude of these $\chi^{\left(1\right)}$, $\chi^{\left(3\right)}$, and $n_{2}$ are typically greater than those reported for previously documented ZnS films [53].

The nonlinear absorption coefficient $\beta_{c}$ of the ZnS and vanadium-doped ZnS layers were estimated based on the equation below [54]:

(17) βc=KcEp12 Fn2Eg3

In this formula, *EP* represents the Kane energy parameter (*EP* = 21 eV), *Kc* is constant (*Kc* = 3100 cm GW-1), and F denotes a function that designates the dispersion of $\beta_{c}$. This function was assessed using the following formula [55]:

(18) F=2hν/Eg−1322hν/Eg5

Figure 10d depicts the relationship between the $\beta_{c}$ of the ZnS and vanadium-doped ZnS and the hv. The graph demonstrates that the values of $\beta_{c}$ rose with the increase in the vanadium content. The incorporation of vanadium can narrow the bandgap in ZnS. This narrowing allows for the absorption of photons with lower energies, which can enhance the nonlinear absorption of the investigated films.

### 3.5. Optoelectrical Parameters

Optical conductivity can be considered a crucial parameter that characterizes the relationship between the induced current density and the strength of the induced electric field across various frequencies. The optical conductivity, $\sigma_{opt}$, of the ZnS and vanadium-doped ZnS layers, was calculated using the equation provided below [56,57]:

(19) $$\sigma_{opt}=\frac{\alpha nc}{4\pi }$$

In this formula, *n* denotes the refractive index of the ZnS and vanadium-doped ZnS layers, while *c* represents the velocity of light.

**Figure 11:**
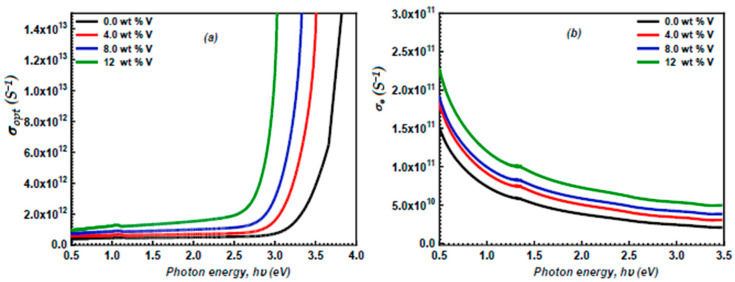
(**a**) The $\sigma_{opt}$ of the ZnS and vanadium-doped ZnS layers in terms of hυ; (**b**) the plot of $\sigma_{e}\;vs.\;h\upsilon$ for the investigated ZnS and vanadium-doped ZnS layers.

Figure 11a illustrates the variation in the optical conductivity, $\sigma_{opt}$, of the undoped and vanadium-doped ZnS films in terms of hv. Increasing the vanadium concentration increases the charge carriers in the films, thereby enhancing the $\sigma_{opt}$ of the undoped and vanadium-doped ZnS layers. Also, the electrical conductivity (*σ_e_*) of the undoped and vanadium-doped ZnS films can be estimated using the following equation [58,59]:

(20) $$\sigma_{e}=\frac{2\sigma_{opt}}{\lambda \alpha }$$

On the other hand, the variation in the electrical conductivity of the ZnS and vanadium-doped ZnS layers with the photon energy is demonstrated in Figure 11b. An increase in the vanadium content improves the electrical conductivity of the ZnS and vanadium-doped ZnS layers. This performance may be related to the enhancement of $\sigma_{opt}$ achieved when boosting the vanadium content.

Meanwhile, optical electronegativity is an important optical parameter that refers to the tendency of an atom to draw an electron from an ionic bond. This method is employed to evaluate the various physicochemical properties of materials. The optical electronegativity ($\eta_{opt}$) and the optical dielectric indices $\varepsilon_{1}$ and $\varepsilon_{2}$ of the spray-deposited ZnS and vanadium-doped ZnS layers were assessed using the formulas below [60,61,62]:

(21) ηopt=An14

(22) $$\varepsilon_{1}=n^{2}-k^{2}$$

(23) $$\varepsilon_{2}=2nk$$

**Figure 12:**
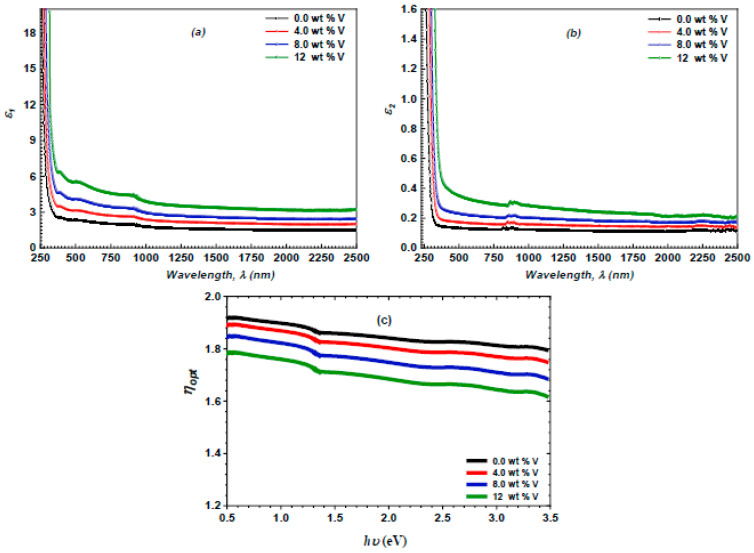
(**a**) The ε_1_ versus λ of the spray-deposited ZnS and vanadium-doped ZnS films; (**b**) the ε_2_ versus λ of the explored films; and (**c**) the $\eta_{opt}$ of the ZnS and vanadium-doped ZnS films versus hυ.

Figure 12a,b demonstrate the fluctuation of ε_1_ and *ε*_2_ in the spray-deposited ZnS and vanadium-doped ZnS layers in terms of λ. The increase in the vanadium content resulted in improved ε_1_ and ε_2_ values for both the ZnS and vanadium-doped ZnS layers. This tendency may be linked to elevated n and k values due to a boost in the vanadium content. Furthermore, Figure 12c illustrates the variation in $\eta_{opt}$ of the ZnS and vanadium-doped ZnS layers with the incident hv. This plot demonstrates that the $\eta_{opt}$ diminishes with an increase in the vanadium content.

The other optoelectrical indices of the vanadium-doped ZnS layers, like the plasma frequency ($\omega_{p}$) and charge carrier concentration (*N*_*opt*_), can be assessed using the equations below [63,64]:

(24) $$\omega_{p}=\sqrt{\frac{e^{2}\;N_{opt}}{\varepsilon_{0}\;\varepsilon_{\infty }\;m^{*}}}$$

(25) $$n^{2}=\varepsilon_{L}-\left(\frac{e^{2}}{4\pi^{2}c^{2}\varepsilon_{0}}\right)\left(\frac{N_{opt}}{m^{*}}\right)\lambda^{2}$$

In these formulas, c refers to the velocity of light, *e* is the electronic charge, and $\varepsilon_{o}$ represents the electric permittivity of free space.

**Figure 13:**
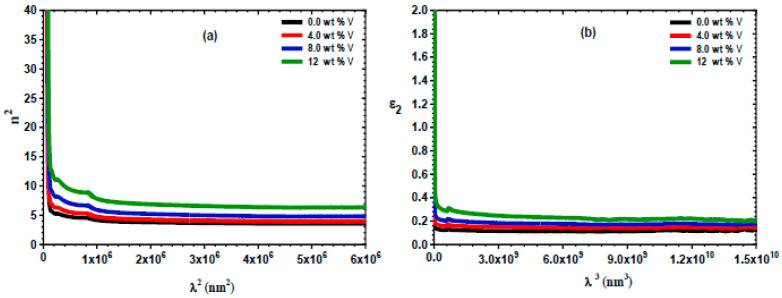
(**a**) The $n^{2}$ of the vanadium-doped ZnS films vs. $\lambda^{2}$ and (**b**) the plot of ε2 vs. $\lambda^{3}$ for the vanadium-doped ZnS films.

Figure 13a demonstrates the variations in $n^{2}\;vs.\;\lambda^{2}$ for the ZnS and vanadium-doped ZnS sheets. The $\left(N_{opt}/m^{*}\right)$ and $\varepsilon_{L}$ were computed from this curve. The $\left(N_{opt}/m^{*}\right)$ and $\varepsilon_{L}$ are documented in Table 3. This table reveals that the $\left(N_{opt}/m^{*}\right)$ and $\varepsilon_{L}$ values were enhanced as the vanadium content increased in the samples. Likewise, the values of $\omega_{p}$ were minimized by increasing the vanadium content.

The relaxation time τ of the ZnS and vanadium-doped ZnS sheets can be estimated by the equation below [65]:

(26) $$\varepsilon_{2}=\frac{1}{4\pi^{3}\varepsilon_{0}}\left(\frac{e^{2}}{c^{3}}\right)\left(\frac{N_{opt}}{m^{*}}\right)\left(\frac{1}{\tau }\right)\lambda^{3\;}$$

Figure 13b illustrates the relationship between *ε*_2_ and *λ*^3^ for the spray-deposited ZnS and vanadium-doped ZnS layers. The τ of the vanadium-doped ZnS samples was evaluated and is documented in Table 3. Increasing the vanadium content minimized the τ of the vanadium-doped ZnS samples.

The optical resistivity (*ρ_opt_*) and optical mobility (*µ_opt_*) of the spray-deposited ZnS and vanadium-doped ZnS layers can be evaluated using the following formulas [66]:

(27) $${µ}_{opt}=\frac{e\tau }{m^{*}}$$

(28) $$\rho_{opt}=\frac{1}{e}\mu_{opt}N_{opt}$$

Table 3 illustrates the values of *µ_opt_* and *ρ_opt_* for the spray-deposited ZnS and vanadium-doped ZnS layers. The increase in the vanadium content improves the values of µopt and *ρ_opt_* in these films.

### 3.6. The Detection of the Semiconductor Type and Charge Compensation Mechanism

In this study, the hot probe method was employed to determine the semiconductor type of the spray-deposited ZnS and vanadium-doped ZnS layers. This procedure involved connecting two probes to a sensitive digital multimeter, with the hot probe linked to the positive terminal and the cold probe to the negative terminal. A positive voltage reading on the multimeter indicates n-type semiconductor behavior, while a negative voltage signifies p-type conductivity [67]. The experimental results consistently showed positive voltage readings for all samples, confirming that both the undoped and vanadium-doped ZnS layers exhibit n-type conductivity. The n-type conductivity observed in the undoped ZnS layers can be attributed to intrinsic defects formed during fabrication, such as sulfur vacancies and zinc interstitials. These defects act as electron donors, contributing free electrons to the conduction band. Sulfur vacancies, for instance, create donor levels within the bandgap, releasing two electrons per vacancy. Similarly, zinc interstitials donate electrons when excess zinc atoms occupy interstitial sites.

On the other hand, the substitution of trivalent vanadium (*V*^3+^) for divalent zinc (Zn^2+^) in V-doped ZnS thin films introduces a charge imbalance, necessitating the use of a compensation mechanism to maintain electrical neutrality. When V^3+^ replaces Zn^2+^, an excess positive charge arises in the lattice. The primary compensation mechanism used involves the formation of zinc vacancies (*Vₐᵧₙ*). For every two *V*^3+^ ions replacing Zn^2+^ ions, one zinc vacancy is created, balancing the charge. This can be expressed as follows:

(29) $${2\;V}^{3+}\overset{ZnS}{\to }{2\;V\;}_{Zn}^{*}+V_{Zn}^{//}$$

Here, ${\;V\;}_{Zn}^{*}$ denotes a V^3+^ ion at a Zn^2+^ site with an effective +1 charge, while $V_{Zn}^{//}$ represents a zinc vacancy with an effective −2 charge. Alternatively, charge compensation may occur through the incorporation of interstitial sulfur or oxygen or co-doping with anionic species like F^−^ or Cl^−^. However, zinc vacancy formation is the most probable mechanism at work due to its low formation energy in the ZnS lattice. This structural defect influences the material’s optical, electrical, and structural properties, modifying the band structure, carrier concentration, and defect-related luminescence of the films, which are critical for optoelectronic applications [68].

## 4. Conclusions

This research work successfully created ZnS and vanadium-doped ZnS layers via cost-effective spray pyrolysis. The XRD study of the investigated layers demonstrates that the layers exhibit a single polycrystalline phase characterized by a hexagonal crystal structure. The analysis of the structural parameters displays that the increase in vanadium content from 4 to 12 wt.% enhanced the values of *εs*, NC, and *δ*, while the crystallite size values were diminished. The FESEM analysis of the pure and vanadium-doped ZnS layers indicates that the examined layers possess a homogeneous surface with a consistent granular structure. The increase in vanadium content minimized the average particle size of the ZnS and vanadium-doped ZnS layers. The linear optical indices of the ZnS and vanadium-doped ZnS layers indicated that an increase in the vanadium ratio enhances the n, k, Eu, and α values of the spray-deposited ZnS and vanadium-doped ZnS layers. The analysis of the optoelectrical indices refers to enhancing the charge carrier’s concentration, the $\sigma_{opt}$, and the $\sigma_{e}$ of the ZnS and vanadium-doped ZnS layers by increasing the vanadium concentration in the analyzed films. Moreover, the increasing of the vanadium concentration in these films enhances the values of the $\chi^{\left(1\right)}$, $\beta_{c},\chi^{\left(3\right)}$, and $n_{2}$ of the ZnS and vanadium-doped ZnS layers. The hot probe experiment indicates a positive voltage in all samples, confirming that the spray-deposited ZnS and vanadium-doped ZnS layers display n-type conductivity.

## Figures and Tables

**Table 1:** The structural parameters of the vanadium-doped ZnS films.

V Content (wt.%)	D (nm)	$\varepsilon_{s}\times 10^{-3}$	$\delta \times 10^{-3}\;({nm}^{-2})$	$N_{C}\times 10^{-3}\;({line/nm}^{3})$
0.0	56.35	0.615	0.314	1.31
4.0	49.67	0.697	0.405	1.98
8.0	44.12	0.785	0.513	2.88
12	40.71	0.851	0.603	3.75

**Table 2:** The optical constants of the undoped and vanadium-doped ZnS layers.

V Content (wt.%)	$E_{u}\;(eV)$	$E_{g}^{dir}\;(eV)$	*E_o_* (eV)	*E_d_* (eV)	*n_o_*	****ε***_s_*	*f*
0.0	0.27	3.74	3.69	9.48	1.89	3.56	34.98
4.0	0.29	3.49	3.25	10.56	2.01	4.06	36.43
8.0	0.31	3.35	2.91	11.34	2.18	4.45	37.31
12	0.34	3.15	2.17	12.76	2.23	5.01	40.57

**Table 3:** The optoelectrical constants of the undoped and vanadium-doped ZnS layers.

V Content (wt.%)	$N_{opt}/m^{*}$ $\left(\times 10^{53}\right)\;{Kg^{-1}m}^{-3}$	$\varepsilon_{L}$	τ $\left(10^{-14}\right)$ s	$\mu_{opt}$ $\left(10^{-3}\right)$ C.s/kg	$\omega_{p}$ $\left(\times 10^{13}\right)$ Hz	$\rho_{opt}$ $\left(\frac{Kg.m^{3}}{C^{2}.s}\right)$
0.0	1.49	4.83	13.93	3.45	4.34	0.18
4.0	1.71	5.04	12.24	5.06	4.07	0.31
8.0	1.84	5.46	9.32	6.35	3.97	0.44
12	2.15	5.80	8.59	7.61	3.67	0.62

## Data Availability

The original contributions presented in this study are included in the article. Further inquiries can be directed to the corresponding author(s).

## References

[1] Saparov B. (2022). Next Generation Thin-Film Solar Absorbers Based on Chalcogenides. Chem. Rev..

[2] Ke D., Zang Z., Zhang Y., Zheng Y., Zhang Y. (2022). The suppressing of excitonic effects in Cu-chalcogenides for solar cell applications. Sol. Energy.

[3] Isotta E., Andrade-Arvizu J., Syafiq U., Jiménez-Arguijo A., Navarro-Güell A., Guc M., Saucedo E., Scardi P. (2022). Towards Low Cost and Sustainable Thin Film Thermoelectric Devices Based on Quaternary Chalcogenides. Adv. Funct. Mater..

[4] Olivier-Fourcade J., Ibanez A., Jumas J.C., Maurin M., Lefebvre I., Lippens P., Lannoo M., Allan G. (1990). Chemical bonding and electronic properties in antimony chalcogenides. J. Solid State Chem..

[5] Kormath Madam Raghupathy R., Wiebeler H., Kühne T.D., Felser C., Mirhosseini H. (2018). Database screening of ternary chalcogenides for p-type transparent conductors. Chem. Mater..

[6] Martínez-Escobar D., Ramachandran M., Sánchez-Juárez A., Rios J.S.N. (2013). Optical and electrical properties of SnSe_2_ and SnSe thin films prepared by spray pyrolysis. Thin Solid Films.

[7] Vinotha K., Jayasutha B., Abel M.J., Vinoth K. (2022). In^3+^-doped CuS thin films: Physicochemical characteristics and photocatalytic property. J. Mater. Sci. Mater. Electron..

[8] Wang L., Tan X., Zhu Q., Dong Z., Wu X., Huang K., Xu J. (2022). The universality applications of MoS_2_@ MnS heterojunction hollow microspheres for univalence organic or multivalence aqueous electrolyte energy storage device. J. Power Sources.

[9] Derbali A., Saidi H., Attaf A., Benamra H., Bouhdjer A., Attaf N., Ezzaouia H., Derbali L., Aida M.S. (2018). Solution flow rate influence on ZnS thin films properties grown by ultrasonic spray for optoelectronic application. J. Semicond..

[10] Afifi H.H., Mahmoud S.A., Ashour A. (1995). Structural study of ZnS thin films prepared by spray pyrolysis. Thin Solid Films.

[11] Mohamed S.H., El-Hagary M., Emam-Ismail M. (2010). Thickness and annealing effects on the optoelectronic properties of ZnS films. J. Phys. D Appl. Phys..

[12] Li Q., Wang C. (2003). Fabrication of wurtzite ZnS nanobelts via simple thermal evaporation. Appl. Phys. Lett..

[13] Simandan I.-D., Sava F., Buruiana A.-T., Burducea I., Becherescu N., Mihai C., Velea A., Galca A.-C. (2021). The effect of the deposition method on the structural and optical properties of ZnS thin films. Coatings.

[14] Elidrissi B., Addou M., Regragui M., Bougrine A., Kachouane A., Bernede J.C. (2001). Structure, composition and optical properties of ZnS thin films prepared by spray pyrolysis. Mater. Chem. Phys..

[15] Echendu O.K., Weerasinghe A.R., Diso D.G., Fauzi F., Dharmadasa I.M. (2013). Characterization of n-type and p-type ZnS thin layers grown by an electrochemical method. J. Electron. Mater..

[16] Khadayeir A.A., Jasim R.I., Jumaah S.H., Habubi N.F., Chiad S.S. (2020). Influence of Substrate Temperature on Physical Properties of Nanostructured ZnS Thin Films. J. Phys. Conf. Ser..

[17] Choubey R.K., Kumar S., Lan C.W. (2014). Shallow chemical bath deposition of ZnS buffer layer for environmentally benign solar cell devices. Adv. Nat. Sci. Nanosci. Nanotechnol..

[18] Vidal J., Vigil O., De Melo O., Lopez N., Zelaya-Angel O. (1999). Influence of NH_3_ concentration and annealing in the properties of chemical bath deposited ZnS films. Mater. Chem. Phys..

[19] Dona J.M., Herrero J. (1994). Process and film characterization of chemical-bath-deposited ZnS thin films. J. Electrochem. Soc..

[20] Oladeji I.O., Chow L. (2005). Synthesis and processing of CdS/ZnS multilayer films for solar cell application. Thin Solid Films.

[21] Dai S., Wang T., Li R., Wang Q., Ma Y., Tian L., Su J., Wang Y., Zhou D., Zhang X. (2018). Preparation and electrical properties of N-doped ZnSnO thin film transistors. J. Alloys Compd..

[22] Akhtar M.S., Malik M.A., Alghamdi Y.G., Ahmad K.S., Riaz S., Naseem S. (2015). Chemical bath deposition of Fe-doped ZnS thin films: Investigations of their ferromagnetic and half-metallic properties. Mater. Sci. Semicond. Process..

[23] Jrad A., Naouai M., Ammar S., Turki-Kamoun N. (2021). Investigation of molybdenum dopant effect on ZnS thin films: Chemical composition, structural, morphological, optical and luminescence surveys. Mater. Sci. Semicond. Process..

[24] Mohamed N.E., Amer M.I., Moustafa S.H., Hashem H., Emam-Ismail M., Shaaban E.R., El-Hagary M. (2024). Improvements of multifunctional spintronic and optoelectronic applications of nanostructured Eu-doped ZnS thin films through magnetic and linear, nonlinear optical investigations. Mater. Chem. Phys..

[25] Mosavi S.M., Kafashan H. (2019). Physical properties of Cd-doped ZnS thin films. Superlattices Microstruct..

[26] Shaili H., Salmani E.M., Essajai R., Beraich M., Battal W., Ouafi M., Elhat A., Rouchdi M., Ez-Zahraouy H., Hassanain N. (2021). Structural, electronic and optical properties of Cu-doped ZnS thin films deposited by the ultrasonic spray method-DFT study. Opt. Quantum Electron..

[27] Jebathew A.J., Karunakaran M., Ade R., Jayram N.D., Ganesh V., Bitla Y., Vinoth S., Algarni H., Yahia I.S. (2021). Optical manipulation of nebulizer spray pyrolysed ZnS thin films for photodetector applications: Effect of Al, Sn and Sb doping. Opt. Mater..

[28] El-Naggar A.M., Heiba Z.K., Kamal A.M., Altowairqi Y., Mohamed M.B. (2022). Enhancing the linear and nonlinear optical properties by ZnS/V-doped polyvinyl alcohol/carboxymethyl cellulose/polyethylene glycol polymeric nanocomposites for optoelectronic applications. J. Mater. Sci. Mater. Electron..

[29] Akl A.A., El Radaf I.M., Hassanien A.S. (2021). An extensive comparative study for microstructural properties and crystal imperfections of Novel sprayed Cu_3_SbSe_3_ Nanoparticle-thin films of different thicknesses. Optik.

[30] Ben Aissa M.A., Khezami L., Taha K., Elamin N., Mustafa B., Al-Ayed A.S., Modwi A. (2021). Yttrium oxide-doped ZnO for effective adsorption of basic fuchsin dye: Equilibrium, kinetics, and mechanism studies. Int. J. Environ. Sci. Technol..

[31] Akl A.A., El Radaf I.M., Hassanien A.S. (2020). Intensive comparative study using X-Ray diffraction for investigating microstructural parameters and crystal defects of the novel nanostructural ZnGa_2_S_4_ thin films. Superlattices Microstruct..

[32] Elamin N., Modwi A., Aissa M.A.B., Taha K.K., Al-Duaij O.K., Yousef T.A. (2021). Fabrication of Cr–ZnO photocatalyst by starch-assisted sol–gel method for photodegradation of congo red under visible light. J. Mater. Sci. Mater. Electron..

[33] Kraidy A.F., El Radaf I.M., Zeinert A., Lahmar A., Pelaiz-Barranco A., Gagou Y. (2024). Optoelectrical properties of the ternary chalcogenide SnSb_2_S_5_ as a new absorber layer for photovoltaic application. J. Phys. D Appl. Phys..

[34] Shkir M., Ganesh V., AlFaify S., Yahia I.S., Zahran H.Y. (2018). Tailoring the linear and nonlinear optical properties of NiO thin films through Cr^3+^ doping. J. Mater. Sci. Mater. Electron..

[35] Tauc J., Grigorovici R., Vancu A. (1966). Optical properties and electronic structure of amorphous germanium. Phys. Status Solidi.

[36] Hassanien A.S., Sharma I., Akl A.A. (2020). Physical and optical properties of a-Ge-Sb-Se-Te bulk and film samples: Refractive index and its association with electronic polarizability of thermally evaporated a-Ge_15−x_Sb_x_Se_50_Te_35_ thin-films. J. Non-Cryst. Solids.

[37] Chandekar K.V., Alkallas F.H., Trabelsi A.B.G., Shkir M., Hakami J., Khan A., Ali H.E., Awwad N.S., AlFaify S. (2022). Improved linear and nonlinear optical properties of PbS thin films synthesized by spray pyrolysis technique for optoelectronics: An effect of Gd^3+^ doping concentrations. Phys. B Condens. Matter.

[38] Hassanien A.S., Sharma I. (2019). Band-gap engineering, conduction and valence band positions of thermally evaporated amorphous Ge_15−x_Sb_x_Se_50_Te_35_ thin films: Influences of Sb upon some optical characterizations and physical parameters. J. Alloys Compd..

[39] Rana M.S., Das S.K., Rahman M.O., Ahmed F., Hossain M.A. (2021). Vanadium doped ZnS nanoparticles: Effect of vanadium concentration on structural, optical and electrical properties. Trans. Electr. Electron. Mater..

[40] Lavanya S., Kumar T.R., Gunavathy K.V., Vibha K., Shkir M., Hakami J., Ali H.E., Ubaidullah M. (2022). A noticeable improvement in opto-electronic properties of nebulizer sprayed In_2_S_3_ thin films for stable-photodetector applications. Micro Nanostructures.

[41] Hassanien A.S., Neffati R., Aly K.A. (2020). Impact of Cd-addition upon optical properties and dispersion parameters of thermally evaporated Cd_x_Zn_1−x_Se films: Discussions on bandgap engineering, conduction and valence band positions. Optik.

[42] Kotbi A., El Radaf I.M., Alaoui I.H., Cantaluppi A., Zeinert A., Lahmar A. (2024). Structural and Optical Characterization of Porous NiV_2_O_6_ Films Synthesized by Nebulizer Spray Pyrolysis for Photodetector Applications. Micromachines.

[43] Jebathew A.J., Karunakaran M., Kumar K.D.A., Valanarasu S., Ganesh V., Shkir M., Yahia I.S., Zahran H.Y., Kathalingam A. (2019). An effect of Gd^3+^ doping on core properties of ZnS thin films prepared by nebulizer spray pyrolysis (NSP) method. Phys. B Condens. Matter.

[44] Lahmar A., Benchaabane A., Aderdour M., Zeinert A., Es-Souni M. (2016). Temperature influence on microstructure and optical properties of TiO_2_–Au thin films. Appl. Phys. A.

[45] Shkir M., Khan A., Imran M., Khan M.A., Zargar R.A., Alshahrani T., Kumar K.D.A., Mohanraj P., Chandekar K.V., AlFaify S. (2022). Spray pyrolysis developed Nd doped Co_3_O_4_ nanostructured thin films and their structural, and opto-nonlinear properties for optoelectronics applications. Opt. Laser Technol..

[46] Al-Zahrani H.Y.S., Alsulami A. (2023). Effects of Cr doping on the structural, optical and electrical characterizations of spray-deposited ZnSnO_3_ thin films. Appl. Phys. A.

[47] Sakli A., Lahmar A., Gamra D., Clin M., Bouchriha H., Lejeune M. (2022). Effect of thermal annealing on microstructure and optical properties of silver-carbon nanocomposite thin films. Mater. Today Proc..

[48] Sharma I., Sharma P., Hassanien A.S. (2022). Optical properties and optoelectrical parameters of the quaternary chalcogenide amorphous Ge_15_Sn_x_S_35−x_Te_50_ films. J. Non. Cryst. Solids.

[49] Khan A., Shkir M., Manthrammel M.A., Ganesh V., Yahia I.S., Ahmed M., El-Toni A.M., Aldalbahi A., Ghaithan H., AlFaify S. (2019). Effect of Gd doping on structural, optical properties, photoluminescence and electrical characteristics of CdS nanoparticles for optoelectronics. Ceram. Int..

[50] El Radaf I.M., Al-Zahrani H.Y.S., Hassanien A.S. (2020). Novel synthesis, structural, linear and nonlinear optical properties of p-type kesterite nanosized Cu_2_MnGeS_4_ thin films. J. Mater. Sci. Mater. Electron..

[51] Paulraj K., Ramaswamy S., Shkir M., Yahia I.S., Hamdy M.S., AlFaify S. (2020). Analysis of neodymium rare earth element doping in PbS films for opto-electronics applications. J. Mater. Sci. Mater. Electron..

[52] Al-Zahrani H.Y.S., Alsulami A. (2023). Structural and optical features of neoteric Ag_2_BaGeS_4_ thin films synthesized by a chemical bath deposition process. J. Mater. Sci. Mater. Electron..

[53] Jebathew A.J., Karunakaran M., Shkir M., Algarni H., AlFaify S., Khan A., Alotaibi N., Alshahrani T. (2021). High sensitive samarium-doped ZnS thin films for photo-detector applications. Opt. Mater..

[54] Alzaid M., Qasem A., Shaaban E.R., Hadia N.M.A. (2020). Extraction of thickness, linear and nonlinear optical parameters of Ge_20+x_Se_80−x_ thin films at normal and slightly inclined light for optoelectronic devices. Opt. Mater..

[55] Hassanien A.S. (2022). Intensive linear and nonlinear optical studies of thermally evaporated amorphous thin Cu-Ge-Se-Te films. J. Non. Cryst. Solids.

[56] Khan M.T., Shkir M., Yahia I.S., Almohammedi A., AlFaify S. (2020). An impact of Cr-doping on physical properties of PbI_2_ thin films facilely deposited by spin coating technique. Superlattices Microstruct..

[57] Alsulami A., Al-Zahrani H.Y.S. (2023). Preparation and characterization of innovative VSbO4 thin films via nebulized spray pyrolysis technique. Opt. Quantum Electron..

[58] Alagarasan D., Hegde S.S., Kumar A., Shanmugavelu B., Murahari P., Ganesan R., Shetty H.D., Naik R., Ubaidullah M., Gupta M. (2023). Influence of La^3+^ doping on nebulizer spray pyrolysed In_2_S_3_ thin film for enhanced photodetector performance. J. Photochem. Photobiol. A Chem..

[59] Hassanien A.S., Sharma I. (2024). Physicochemical, optical, and dielectric studies of physically vapor deposited amorphous thin Cu_25−x_ (ZnGe)_25−x_Se_50+2x_ films. J. Non. Cryst. Solids.

[60] Berra S., Mahroug A., Hamrit S., Ahmad M., Zoukel A., Berri S., Selmi N. (2022). Experimental and DFT study of structural and optical properties of Ni-doped ZnO nanofiber thin films for optoelectronic applications. Opt. Mater..

[61] Ahmoum H., Chelvanathan P., Su’ait M.S., Boughrara M., Li G., Al-Waeli A.H.A., Sopian K., Kerouad M., Amin N. (2020). Impact of preheating environment on microstructural and optoelectronic properties of Cu_2_ZnSnS_4_ (CZTS) thin films deposited by spin-coating. Superlattices Microstruct..

[62] Alsulami A., Alsalme A. (2023). Synthesis, Structural, Optical and Optoelectrical Properties of the Chemically Deposited Cu_2_BaGeS_4_ Thin Films. ECS J. Solid State Sci. Technol..

[63] Hossain M.F., Shah M.A.H., Islam M.A., Hossain M.S. (2021). Transparent conducting SnO_2_ thin films synthesized by nebulized spray pyrolysis technique: Impact of Sb doping on the different physical properties. Mater. Sci. Semicond. Process..

[64] Hassanien A.S., El Radaf I.M. (2023). Effectiveness of Sn-addition on optical properties and physicochemical parameters of Sn_x_Sb_2−x_Se_3_ thin films. Mater. Chem. Phys..

[65] Shaaban E.R., Hassaan M.Y., Moustafa M.G., Qasem A., Ali G.A.M. (2019). Optical constants, dispersion parameters and non-linearity of different thickness of As_40_S_45_Se_15_ thin films for optoelectronic applications. Optik.

[66] Hassanien A.S., Sharma I., Aly K.A. (2021). Linear and nonlinear optical studies of thermally evaporated chalcogenide a-Pb-Se-Ge thin films. Phys. B Condens. Matter.

[67] Golan G., Axelevitch A., Gorenstein B., Manevych V. (2006). Hot-probe method for evaluation of impurities concentration in semiconductors. Microelectron. J..

[68] Jubeer E.M., Manthrammel M.A., Subha P.A., Shkir M., Biju K.P., AlFaify S.A. (2023). Defect engineering for enhanced optical and photocatalytic properties of ZnS nanoparticles synthesized by hydrothermal method. Sci. Rep..

